# Hope and trust: Public attitudes toward mass COVID-19 testing programs in Guangzhou, China

**DOI:** 10.3389/fpsyg.2022.972398

**Published:** 2022-09-15

**Authors:** Xuanxuan Tan

**Affiliations:** Department of Cultural and Religious Studies, Faculty of Arts, The Chinese University of Hong Kong, Hong Kong, Hong Kong SAR, China

**Keywords:** COVID-19, hope, trust, mass testing, biomedicine, China, risk responses, social psychology

## Abstract

Mass testing is one COVID-19 pandemic response strategy. The effect of population-wide testing programs is influenced by public attitudes toward COVID-19 viral tests. However, the public’s attitudes toward mass testing and related factors in mainland China are not adequately understood. This study focuses on pandemic responses during the first wave of the Delta variant outbreak in southern China and explores how residents responded to population-wide mass COVID-19 testing programs. The research relies on data collected from short videos recording residents’ experiences of being in lockdown, media reports, and semi-structured interviews. Thematic analysis was used to analyze the data, and four themes emerged: public attitudes toward mass testing, the technology of viral tests, policy and governance, and cultural practices. The study finds that residents actively participated in mass testing campaigns as mass viral tests are associated with hope and trust. The Reverse Transcription–Polymerase Chain Reaction (RT-PCR), negative test results, lockdown policies, waiting times, medical staff, and media representations are all actors that assemble and mobilize hope and trust. The research reveals some critical factors influencing people’s attitudes toward mass testing policies in response to COVID-19 and provides practical suggestions for public health professionals in rolling out effective population-wide mass COVID-19 testing.

## Introduction

The Coronavirus Disease 2019 (COVID-19) pandemic poses risks and uncertainty for the entire world. Understanding the character of pre-symptomatic and asymptomatic transmission among patients with COVID-19, primarily diagnosed through the detection of viral RNA *via* Reverse Transcription–Polymerase Chain Reaction (RT-PCR), is critical for guiding infection control policy. Viral testing of targeted populations or massive viral tests usually involves conducting a large volume of RT-PCR tests. Mass testing can identify asymptomatic/pre-symptomatic cases, contain community virus transmission, relieve pressure on the health care system (European Centre for Disease Prevention and Control, 2020), and improve the surveillance of viral mutations (Peeling et al., 2020). It also increases the effectiveness of contact tracing (Clark et al., 2021) and reduces the number of health workers in quarantine facilities, promoting economic recovery (Atkeson et al., 2020). Widespread testing programs have been rolled out to combat the pandemic in China (Pan et al., 2021; Xin et al., 2022), England (Iacobucci, 2020), Luxembourg (Luxembourg Institute of Health, 2020), Slovakia (Holt, 2020), Denmark, France, and Lithuania (European Centre for Disease Prevention and Control, 2020). Germany and South Korea employed massive viral testing to detect infections in severely affected communities (Dighe et al., 2020).

The efficacy of mass COVID-19 testing is influenced by multiple factors, including public participation, epidemiological situation, costs, backup resources, technical practicability, resource availability, contact tracing and quarantine ability, testing difficulties, potential rate of false positivity, and information release (European Centre for Disease Prevention and Control, 2020). A good understanding of the public’s attitude toward mass testing and related factors is vital for rolling out effective population-wide mass testing. In countries and areas such as the United States (Clark et al., 2021) and Hong Kong (Xin et al., 2022), public acceptance of massive viral testing programs is not high. Representations of COVID-19, perceived vulnerability to COVID-19, assumed effects of the mass testing campaign, and trust in the government’s pandemic control measures are multi-dimensional factors that influence people’s participation in mass testing in Hong Kong (Xin et al., 2022). Although massive viral testing programs are one of the dominant anti-pandemic measures used in mainland China, public attitudes toward massive viral testing and the multi-dimensional factors that influence these attitudes have not been adequately investigated. Thus, this research asks: (1) What are the public attitudes toward massive viral testing campaigns in China? (2) What factors and actors shape people’s attitudes toward mass testing? and (3) How do these actors shape people’s attitudes?

## Materials and methods

### Research context, settings, and design

The research context was the Guangzhou Delta variant outbreak. The Guangzhou Delta variant outbreak was the first wave of an outbreak caused by the Delta variant in China. Guangzhou is the capital city of Guangdong province in the southern part of China. It is one of the most developed metropolises in China and is close to Shenzhen and Hong Kong. The variant caused 153 infection cases in Guangdong province, most of which (146) were in Guangzhou. The outbreak also affected cities near Guangzhou, such as Dongguan, Shenzhen, Zhongshan, Zhuhai, and Jiangmen. The first case was detected on 20 May 2021. One month later, the outbreak was successfully controlled. Guangzhou organized a massive viral testing campaign for 18.67 million permanent residents during the Delta variant outbreak in June 2021, the largest scale of mass testing in a global city (Xinhua Net, 2021). On 5 June 2021, all 11 districts in Guangzhou started massive nucleic acid testing, and 27.9855 million nucleic acid samples had been collected by 7 June 2021. Several cities influenced by the Delta variant outbreak also conducted massive viral testing to respond to the pandemic. Fangcun, an area in the Liwan District, was the most severely affected area. All nine streets in Fangcun, with a population of more than 187,700, were locked down on 3 June 2021. Traffic control was implemented in these areas, and local governments and communities strictly managed residents’ activities. The local government employed the multi-level approach for prevention and control. The management of different areas in Fangcun were subjected to change according to the outbreak situation in the areas. On 17 June 2021, the local government adjusted the lockdown policy and allowed people in the six streets of the lockdown areas leave home with a permit. Residents not including those living in areas undergone closed management could go shopping and move in Fangcun. Several bus lines were reopened while residents and cars could not leave Fangcun. On 24 June 2021, all nine streets were unlocked, and residents could leave Fangcun. Multiple mass screenings were rolled out to detect infection cases in lockdown districts. Residents in quarantine zones such as Hailong Street in Fangcun underwent nucleic acid testing eight times during the Fangcun lockdown.

The author examined public attitudes toward mass testing and multi-dimensional factors during a field trip to four cities in Guangdong provinces including Guangzhou, Shenzhen, Dongguan, and Jiangmen from June 2021 to August 2021. This study employed a qualitative approach to examine how people respond to population-wide testing programs. Thematic analysis was used to analyze the data collected from semi-structured interviews with residents who experienced the Delta variant outbreak, short videos recording residents’ experiences of being in lockdown, and media reports.

### Data collection

Thirty-four semi-structured interviews were conducted for this research. Specifically, 29 residents living in Guangdong province who had experienced or were influenced by the Guangzhou Delta variant outbreak were interviewed. These participants were invited to talk about their reaction to the pandemic and answered questions about how they experienced, responded to, and thought about the massive viral tests. To better understand medical knowledge about RT-PCR and how this technology was employed through mass testing during the Guangzhou outbreak, interviews were also conducted with five frontline medical workers, medical researchers, and public health professionals familiar with the public health policy, RT-PCR, and mass testing implemented as part of China’s pandemic response. Interviews lasting between 30 min and 2 h were conducted face-to-face or *via* online conversations. The interviews were audiotaped and transcribed verbatim. Pseudonyms were used to protect identities.

Some residents in quarantined areas of Fangcun recorded their everyday lives during the lockdown, producing short videos and uploading them to social media platforms from May to June 2021. For this study, 147 videos uploaded by seven residents during the Guangzhou Delta variant outbreak were collected to investigate how residents in lockdown areas experienced and thought about massive viral tests. These videos were collected from one of China’s most popular video platforms, Watermelon Video. Media reports, official documents, and online discussions introducing public and local governments’ pandemic responses during the outbreak were also collected to better understand public responses to massive testing and the factors shaping these attitudes. These media reports and policy documents were produced by official media such as Guangzhou Daily and other entities in Guangdong province. This study also collected discussions posted on WeChat, a Chinese social media platform, in which residents living in areas affected by the outbreak shared their viral testing experiences.

### Participants

To recruit participants for the semi-structured interviews, an advertisement was posted on WeChat. It invited residents living in Guangdong province who had participated in mass testing during the Guangzhou Delta variant outbreak to talk about their own reaction to the pandemic and their attitudes toward and perceptions of massive viral tests. Medical and public health professionals were also contacted and interviewed based on colleagues’ and interviewees’ recommendations to collect medical knowledge about viral tests and how they were employed during the Guangzhou outbreak. Table 1 shows the participants’ demographic characteristics. Danny, May, Moe, Chen, and Martian are medical and public health professionals. The short videos produced by residents in lockdown areas are considered secondary data, and these residents’ demographic information was also considered. Zoe, San, Mike, Lily, George, Jimmy, and Zine are producers of the short videos living in Fangcun. Since the users’ identities were anonymous on the video platform, these residents’ ages were difficult to determine. However, their genders, lockdown experience during the Guangzhou outbreak, and places of residence could be identified by analyzing video content.

**Table 1:** Demographic information of participants.

Pseudonyms	Age range (years)	Gender	Place of residence	Lockdown experience
Way	18–30	F	Guangzhou	No
Canny	18–30	M	Guangzhou	Yes
Eric	30–40	M	Guangzhou	Yes
May	18–30	F	Guangzhou	No
Tao	18–30	M	Guangzhou	Yes
Fly	18–30	M	Dongguan	No
Lucas	18–30	M	Guangzhou	No
Lu	18–30	M	Guangzhou	No
Sally	18–30	F	Guangzhou	No
Yann	18–30	F	Dongguan	No
Li	18–30	M	Guangzhou	No
Peter	30–40	M	Shenzhen	No
Ping	30–40	M	Shenzhen	No
Jim	18–30	F	Guangzhou	No
Shen	18–30	F	Guangzhou	No
Lax	30–40	F	Guangzhou	Yes
Winne	18–30	F	Guangzhou	No
Zizi	18–30	F	Guangzhou	Yes
Fancy	30–40	F	Guangzhou	Yes
David	30–40	M	Guangzhou	Yes
Sunny	50–60	F	Jiangmen	No
Tommy	18–30	M	Dongguan	No
Jane	50–60	F	Dongguan	No
Juliet	50–60	F	Dongguan	No
Nancy	18–30	F	Guangzhou	No
Zinn	50–60	F	Zhongshan	No
Cherry	18–30	F	Guangzhou	Yes
Penny	Undisclosed	M	Guangzhou	Yes
Mandy	Undisclosed	M	Dongguan	No
Danny	Undisclosed	M	Guangzhou	Undisclosed
May	18–30	F	Jiangmen	Undisclosed
Moe	Undisclosed	M	Dongguan	Undisclosed
Chen	30–40	M	Hong Kong	No
Martian	18–30	M	Hong Kong	No
Zoe	Unknown	F	Guangzhou	Yes
San	Unknown	F	Guangzhou	Yes
Mike	Unknown	M	Guangzhou	Yes
Lily	Unknown	F	Guangzhou	Yes
George	Unknown	M	Guangzhou	Yes
Jimmy	Unknown	M	Guangzhou	Yes
Zine	Unknown	F	Guangzhou	Yes

### Theoretical perspective

The present study explores public attitudes toward mass testing and the multi-dimensional factors related to participation in testing from the perspective of Science and Technology Studies (STS). It employs the conceptual emphases of actor–network theory (ANT) within STS as a new way to examine public attitudes toward anti-pandemic measures and technologies used during the pandemic. ANT provides a package of tools to explain society by investigating relations among concepts rather than focusing on essential definitions, stable entities, or unquestioned categories. It challenges dominant dualisms related to social versus natural explanations (Krarup and Blokm, 2011). This research selectively adopts two conceptual highlights of ANT to guide data collection and analysis. The first involves shedding light on the practices and roles of both human and non-human actors in society. Humans are situated in the broader network constituted by human and non-human actors. ANT posits that people, emotions, policies, guidelines, technologies (e.g., viral test technology and laboratory work), and media representations affect the application of biomedicine. The second is the “sociology of associations,” which understands the social as the continuing heterogeneous associations of changing relations between human and non-human actors (Callon, 1999; Latour, 2005). Entanglement, an ANT term, describes the heterogeneous and inter-related environment constituting people, things, and thoughts. This term is useful when analyzing the dynamic interplay, modes, and assemblages generated by human and non-human actants, which produce practices, knowledge, and technologies (Callon, 1999). These conceptual emphases inspire this study’s interest in how human and non-human actors and their associations in society shape people’s attitudes toward mass testing. Specifically, this research regards mass testing as an assemblage of material forms and networks of significance, in which not only viral test kits and human movement but also scientific, cultural, and political meanings are assembled (Latour, 2005). As a complex network, the nucleic acid test includes the residents being tested, RT-PCR, medical staff, policy documents, media representation, time spent on the test, volunteers, knowledge about the test, the digital platforms releasing the test results, and so on. This research analyzes how these human and non-human actors and their entanglements in the network of massive viral testing shape people’s attitudes toward mass testing.

### Data analysis

The data were analyzed and interpreted using thematic analysis (Braun and Clarke, 2006). This method employs an inductive approach to identify themes. Following the thematic analysis protocol, the data were familiarized, and codes generated from the initial coding process were categorized into themes across the data (Braun and Clarke, 2006). The themes were named and defined. More specifically, they were organized with the two conceptual emphases of ANT in mind. Human and non-human actors and their associations were identified from the preliminary codes before categorizing them into various themes.

## Results

The findings of the thematic analysis on public attitudes toward mass testing and factors influencing these attitudes were organized into four emerging themes: public attitudes toward mass testing, the technology of viral tests, policy and governance, and cultural practices. Table 2 shows the coding tree of the thematic analysis. The first theme suggests that residents display positive emotions, such as hope and trust, when participating in mass screenings. The next three themes illustrate how various human and non-human actors shape people’s attitudes. Specifically, the second theme reveals how people’s perceptions and understandings of the technology of massive viral tests, including RT-PCR, and of negative test results are associated with certainty and comfort, which further facilitate individuals’ positive responses to mass testing. The third theme, exploring the political factors shaping public attitudes, shows how lockdown policies and the organization of and participation in free population-wide testing programs influence people’s attitudes toward mass viral tests. The fourth theme describes how the media culture of viral testing reflects and shapes residents’ perception of mass screenings.

**Table 2:** Coding tree of thematic analysis.

Theme	Sub-themes
Public attitudes toward mass testing	Hope
	Trust
The technology of viral tests	RT-PCR
	Negative test results
Policy and governance	Lockdown policy
	Mass testing process
Cultural practices	Media representations

### Theme 1: Public attitudes toward mass testing

#### Sub-theme 1.1: Hope

Public attitudes toward mass testing were positive as the nucleic acid test was associated with a hope about lifting the lockdown and the end of the quarantine life as well as the outbreak. Zoe, a resident living in Fangcun, argued that the frequency of nucleic acid testing was positively associated with lifting the lockdown. She titled one of her posts, “It is another day at home and work in Guangzhou. The third nucleic acid test is done, and the day of unlocking is not far away” when sharing her experience participating in mass testing (short video, Rec 00042). When preparing for the eighth nucleic acid testing, she said, “Today is June 23, we are doing the eighth nucleic acid testing, and we will lift the lockdown” (short video, Rec 00025). For Zoe, the nucleic acid test brought hope of ending the lockdown. Mike, another resident going through the Fangcun lockdown, shared an opinion similar to Zoe’s, saying, “Today we conduct the last round of massive nucleic acid testing, in groups of five. The result of today’s nucleic acid test is significant. If the result is negative, the lockdown of Fangcun will be lifted” (short video, Rec 00059). Mike highlighted the great significance of the last round of massive nucleic acid tests, and he even predicted that a negative result meant the end of quarantine life. Another resident, Lily, also conveyed her hope of ending the outbreak with the subtitle, “Tomorrow is the sixth nucleic acid test. Hope the pandemic will end soon, and the day of lifting the lockdown is approaching” (short video, Rec 00084). Although Lily did not argue that the nucleic acid test would bring the end of the outbreak, associating the hope of ending the pandemic and lifting the lockdown with mass testing indicates that her attitude toward massive viral tests was positive.

#### Sub-theme 1.2: Trust

Expressing trust in the government when participating in massive nucleic acid testing demonstrated that residents’ positive attitudes toward massive viral tests. Residents in lockdown areas used music conveying positive feelings such as hope, confidence, and fighting to represent massive nucleic acid test campaigns. George appropriated a song called “Your Answer,” sung by the singer A Rong, with the lyrics, “Go forward bravely/The light of dawn/Will cross the darkness/Break all fear I can/Find the answer” in his short video of mass testing (short video, Rec 00108). The background music and lyrics, such as “Go forward bravely” and “Will cross the darkness,” constructed a particular meaning in relation to mass testing, associating it with positive feelings and implying that massive viral tests could help Guangzhou win the war against the outbreak. In non-quarantine areas, massive nucleic acid test campaigns were associated with residents’ trust in the government. Resident Sally argued that the Guangzhou government could effectively contain the outbreak as four rounds of massive nucleic acid testing had been conducted since the first infection was reported. In other words, the massive test increased her trust in the government’s pandemic response. People engaged in massive viral testing programs and shared their experiences *via* social media platforms. Resident Fly, a young programmer, said several of his friends shared their experiences *via* WeChat. His friends posted updates using words such as “fighting” and admiring the medical staff’s hard work in rolling out mass testing to express positive attitudes toward the testing. Similarly, Li shared a post on WeChat after participating in a mass screening, saying, “Tonight is sleepless. Thanks to the volunteers and the staff at the testing points. Thumbs up. Thumbs up. The mass testing was going smoothly. Great! Guangzhou, fighting!” These posts indicate that residents demonstrated positive attitudes toward massive viral tests.

### Theme 2: The technology of viral tests

#### Sub-theme 2.1: RT-PCR

The technological certainty of RT-PCR brought people certainty and comfort and it further facilitated individuals’ trust in the government’s pandemic response. Uncertainty refers to an unknowable situation: “We know that we do not know, but that is almost all we know: there is no better definition of uncertainty” (MacPhail, 2014). As the opposite of uncertainty, certainty is related to a knowable situation or a factor that makes an unknowable situation knowable. Certainty in residents’ perspectives meant feeling at ease, reassuring oneself, and proving one’s health status, that is, that one has not infected others. Medical representations of disease are situated in a particular social, cultural, and political context. The medical world, society, media, and patients’ communities all have different understandings of the same disease (Engelmann, 2018). The medical world and the public also have different understandings of biomedicine, in this case RT-PCR. In medicine and epidemiology, RT-PCR is not 100% accurate. The challenges of RT-PCR remain “throughout the entire analytical process, from the collection and treatment of specimens to the amplification and detection of viral RNA and the validation of clinical sensitivity and specificity” (Feng et al., 2020). However, in comparison to scientists, the study’s informants had different knowledge about RT-PCR. They believed that RT-PCR generated certainty as it made SARS-CoV2 visible. It identified infected and uninfected bodies by generating positive or negative results, thus making the virus visible and providing evidence for public health professionals to identify infections and delineate restricted and quarantined areas. Thus, people trusted the ability of RT-PCR to bring them certainty.

Amplification, one of the processes RT-PCR undergoes in the laboratory, involves using TaqMan probes, which are oligonucleotides used to “capture” the target sequences identifying SARS-CoV2 and help the PCR machine produce accurate real-time RT-PCR results. Amplification also involves the proliferation of specific DNA targets so that SARS-CoV2 can be easier “seen” and “detected.” The results are represented by the CT value. If the CT values ≥ 35, the result is negative and vice versa. TaqMan probes, the PCR machine, and the CT matrix curve, as non-human actors, make the virus visible to humans and generate certainty. In this sense, viruses becoming visible *via* amplification are informants at the center of scientific research and global pandemic policymaking (MacPhail, 2014). By seeing (positive result) or not seeing (negative result) the virus, it becomes evidence of infection or not. Accordingly, the nucleic acid test made people feel at ease as they could get evidence of good health and thus certainty. Informants who participated in massive nucleic acid test campaigns said that nucleic acid tests made them feel relieved by ensuring they had not contracted COVID-9. Fly had not had a nucleic acid test for a long time leading up to the Guangzhou outbreak. Although he had not visited areas in which infections had been reported, he went to work every day and could not be certain of whether he was healthy or not. The negative RT-PCR result made him feel at ease by proving he was not an infected body. Thus, the nucleic acid test comforted people and diminished uncertainty.

Residents still said that RT-PCR generated certainty for them even after learning that RT-PCR is not 100% accurate. At the same time, since RT-PCR is not 100% accurate, multiple rounds of massive viral tests are necessary. Sally knew that sampling and viral load in human bodies both influence the efficacy of RT-PCR. She emphasized that the virus is more difficult to detect in patients for whom the virus is in the incubation period than for patients with the virus in the onset period. This pathological feature explains why some people tested negative 11 times before testing positive in the 12th viral test. Against this backdrop, Sally argued, “multiple viral tests are necessary and can make me feel at ease.”

The technological certainty provided by the nucleic acid test also granted individuals certainty and comfort on an emotional and moral level. Besides actively participating in massive nucleic acid test campaigns, Sally also closely monitored her health and was willing to take self-financed nucleic acid tests in the hospital. One day, she felt she might be infected with COVID-19 as she had a throat sore and a low-grade fever. Sally visited a hospital near her workplace and requested a doctor give her a nucleic acid test. The doctor said it was unnecessary as she had a normal body temperature, but Sally insisted on having the test; finally, the doctor arranged a nucleic acid test for her. Sally intended to gain comfort from the nucleic acid test because it made her feel at ease on a moral level. “When you know you are healthy, you cannot infect other people or hurt others; this is always a way to make you feel at ease,” she said.

#### Sub-theme 2.2: Negative test results

Negative test results of RT-PCR granting residents’ certainty were associated with individuals’ positive responses to mass screening. Sally was eager to seek certainty through multiple rounds of nucleic acid tests and said the negative result indicated that “the SARS-CoV2 has not invaded you and you are a healthy body.” Peter agreed that the negative results of multiple rounds of nucleic acid tests meant you were very likely to be healthy, and that it was necessary to conduct multiple rounds of massive nucleic acid tests. He said, “You can see that it is challenging to trace the source of infection. A person visits many places and contacts different residents every day. It is hard to find infected people one by one. Only through this large-scale nucleic acid test can we find the infected person. Multiple rounds of massive nucleic acid tests are also a safer approach. If you do it several times and the results are negative, there is a high probability that you will be fine in the end.” Nucleic acid tests, in bringing people certainty and comfort, became the material representation and source of people’s trust in government. For instance, Sally believed massive nucleic acid tests were necessary and practical. When the Guangzhou government conducted four rounds of massive nucleic acid tests, she believed the local government was using an effective measure to detect infection cases and had trust and confidence in Guangzhou’s pandemic response.

### Theme 3: Policy and governance

#### Sub-theme 3.1: Lockdown policy

The lockdown policy highlighting that mass testing was an essential anti-pandemic measure employed in lockdown areas shaped residents’ attitudes toward mass testing. China has adopted a “zero infection policy” and a “dynamic zero infection policy” to respond to the COVID-19 pandemic. These policies are based on a strategy of visualizing infections, detecting infections, and mapping out the possibility of infections and viral transmission. These policies have provided guidelines for conducting massive nucleic acid tests and imposing lockdowns. Mass testing is implemented to measure the prevalence of COVID-19 infection in communities, classify areas into different levels of risk (Studdert and Hall, 2020), detect infection cases for cutting off the chain of virus transmission (Xinhua Net, 2021), and evaluate the risk status of quarantine zones for determining when lockdowns can be lifted (China Daily, 2021). In this sense, the massive nucleic acid tests, from residents’ perspective, signaled the lifting of the lockdown and produced some hope of ending quarantine life. Thus, residents such as Zoe, Lily, and Mike, while obeying quarantine measures, expressed hope when taking the nucleic acid tests in their short videos. Lily recorded her experience of having the seventh nucleic acid test and at the end of the video (Rec 00081), she added the text “Hope the lifting of the lockdown is approaching as soon as possible.” She quoted a message received from the local government about notification of mass testing to introduce the video: “Warm reminder: Dear citizens, hello! Today (June 16), Liwan District is carrying out a new round of nucleic acid testing for all residents in the lockdown areas. Please follow the guidelines released by the residents’ committee and participate in mass testing at the testing point before 17:00 today, wearing a mask. Please keep social distancing, reduce social contact, follow the testing instructions, leave the testing points as soon as possible after nucleic acid sampling, and go back home to practice domestic health management. Let us fight the pandemic together with hope. Thank you for your support and cooperation!” This message indicates mass testing is a vital anti-pandemic measure employed in the lockdown areas and is associated with the hope of ending the outbreak. Lily quoted this message to introduce her experience of participating in mass testing suggesting she believed mass testing signals the ending of the outbreak and thus expressed the hope of the lifting of the lockdown at the end of the video.

#### Sub-theme 3.2: Mass testing process

Time spent on viral tests, special arrangements, and frontline medical workers were human and non-human actors in the mass testing process which influenced people’s attitudes toward mass screenings. The Chinese National Health Commission requires cities to complete mass testing of their populations within 3 days (Bao, 2021). This population-based testing program is a challenge for local governments. Residents expressed positive attitudes toward mass testing as the process of multiple rounds of massive nucleic acid tests demonstrated the government’s capability of responding to the pandemic; further, it made people trust that the government was effectively fighting the pandemic. Fly argued that massive viral tests were persuasive evidence of a local government’s emergency response capability. From his perspective, massive nucleic acid tests cost a large amount of money, consumed medical supplies such as test kits and protective suits, and mobilized hundreds of medical staff, members of the Chinese Communist Party, grassroots officers, and volunteers in 1 or 2 days. If a government could effectively and orderly arrange massive nucleic acid tests for several million people within days, it was powerful and capable, and thus it deserved people’s trust, positive comments, and collaboration.

The massive testing involved several steps. First, residents received announcements of mass testing which listed the locations of testing points, timeslots, covered populations, and the overall testing guidance. Then, residents went to the nearly testing points and showed health codes to medical staff or officials before entering the testing points. To identify people being tested, residents needed to use their mobile phones to scan a QR code to visit a mini-program called “Yue Hesuan” and uploaded information such as names, phone numbers, and identity card numbers in the mini-program. A QR code would be generated automatically in the mini-program and shown on the residents’ mobile phone screens after individuals submitted the information. Frontline medical workers at the testing points then scanned the QR code to identify the identity of the individuals being tested before the sampling process. The testing results would be uploaded to the health code system and residents could check them up on their mobile phones. People with disabilities, the elderly, children, and pregnant women could receive special arrangements which help them finish the sampling process. Specifically, time spent on viral tests, special arrangements, and frontline medical workers were human and non-human actors that shape people’s attitudes toward massive testing campaigns. Residents praised frontline medical workers, expressed hope and trust, and accepted the massive viral test as the time spent on the test was short. They highlighted the excellent management during massive viral testing and focused on waiting times, volunteers, and medical workers. Shan, working in Guangzhou, said, “It was very hot, over 30°C. The weather was not well. I had participated in three rounds of large-scale nucleic acid tests in four days. The medical staff were working hard!” Sisi uploaded a photo of the viral testing site and said, “Greetings to the great medical staff,” with a national flag emoji on 7 June 2021. Mike said, “the process of nucleic acid detection is fast. It can be completed in about 20 min.” He also described the “super loving, funny, and responsible nurses,” praised frontline medical workers’ efforts in organizing mass testing, and argued that “we should obey pandemic prevention and control measures” (short video, Rec 00069). Thus, massive viral tests’ sampling speed reflected medical workers’ effort, highlighting local governments’ organizational capacity. Additionally, special arrangements for massive nucleic acid tests targeting vulnerable groups, such as “no queuing, priority detection,” shape individual attitudes toward mass testing. Residents with disabilities argued that the special arrangements indicated the local government’s capacity to provide care and help to vulnerable individuals. For example, Jimmy is a young person suffering from Amyotrophic Lateral Sclerosis (ALS) who went through the Fangcun lockdown. He expressed his appreciation for the local government because he did not need to wait in a line and received help from policemen during the testing (short video, Rec 00186).

### Theme 4: Cultural practices

#### Sub-theme 4.1: Media representations

This sub-theme showed how media representations generating cultural meanings about free-population wide testing programs mapped out residents’ positive attitudes toward RT-PCR and further influenced their participations in mass screenings. Media representations connected to power and knowledge influence people’s responses and understanding of the outbreak (Keck et al., 2019). It has been argued that the discourses of an epidemic help to map out the power relations, knowledge, and credibility systems and determine the reality of the epidemic and the disease (Treichler, 1999). Similarly, as an outbreak narrative, media representations of massive viral tests have consequences. The nucleic acid test operates as a cultural object that constructs and generates hope and trust *via* media representation on news and social media platforms. Media reports about mass testing used the negative results of nucleic acid tests to represent massive nucleic acid testing. Official media used titles such as “Nucleic acid test result of all residents in Guangzhou Liwan North Area: 532,200 people, 100% negative!” (Xin Kuai Newspaper, 13 June 2021) to report on massive nucleic acid testing. The “100% negative” phrase highlights that massive nucleic acid tests provided good results, and that 532,200 people were healthy. Related media reports gave the public a sense of certainty, proved the government was trustworthy, and generated the hope of ending the epidemic.

The roles of medical staff and volunteers at massive nucleic acid test sites were highlighted in media representations to generate hope about winning the war against the outbreak and trust in the local government: “Nice to have you! The local epidemic broke out in Guangdong, and the medical staff will be dispatched again!” (Official account of Guangdong Provincial Health Commission, May 27, 2021) and “After 33 days, high-risk areas have reappeared across the country! Hundreds of doctors and nurses are collecting samples in the rain. Guangzhou, come on!” (Health Circle, 2 June 2020). Official media use these titles to highlight medical workers’ efforts and sacrifices and to construct them as ordinary heroes who are bravely fighting the outbreak. These media representations are similar to those used during the SARS outbreak in 2003. Both SARS and COVID-19 were constructed as wars, with people relying on mass mobilization, collectivism, and patriotism (Lu, 2008). This representation strategy shifts the public’s attention from the outbreak and massive viral tests to the human actors who conduct the testing. More importantly, it employs a heroic narrative and discourses about collectivism to construct an image of medical workers as professional and selfless, thus implying that ordinary people should trust in them and in the local government and have confidence and hope that they are winning the war against COVID-19. These media representations influence people’s attitudes toward mass viral tests. May, a resident of Guangzhou, said her neighborhood forwarded news about mass testing and media reports highlighting medical workers’ efforts and sacrifices in WeChat group to advocate other people actively engaged in massive viral tests. Her neighborhood argued they should not betray their hard work but instead actively engage in massive viral tests.

## Discussion

Negative emotions are a typical response to the pandemic. People feel fearful (Commodari and La Rosa, 2020; Consolo et al., 2020; Yang et al., 2020), depressed (Li et al., 2020), stressed (Jones et al., 2021), and anxious (Consolo et al., 2020; Li et al., 2020; Chen et al., 2021). However, this research reveals that the public has adopted positive attitudes toward mass screenings, suggesting that emotional responses to the outbreak can also be positive. This research finding supports current studies arguing that positive emotions have played a significant role in China’s response to the pandemic (de Kloet et al., 2020, 2021; Ni et al., 2020; Litzinger and Ni, 2021) and provides evidence regarding the role of this positive psychology during the pandemic. These positive emotions can be regarded as risk response strategies. Weighing pros and cons, making calculations, trust, intuition, emotion, belief, hope, and faith are strategies for managing risk and uncertainty (Zinn, 2008, 2016). Trust and emotions are in-between strategies, and hope is a non-rational strategy in risk management (Zinn, 2016). This study shows how some residents employ these in-between and non-rational strategies to respond to the uncertain outbreak. In other words, hope and trust are tools to help people deal with an uncertain outbreak and to tolerate psychological and economic pressure during the lockdown. Further research could examine the interplay of positive emotions and negative psychological conditions to map out the complexity of social psychology during the pandemic.

Different actors in the network of massive viral testing, such as RT-PCR, medical staff, negative test results, and queue times, shape people’s attitudes toward massive viral tests and influence their emotions, such as hope and trust. In other words, massive viral tests contribute to the proliferation of people’s hope and trust during the outbreak. These findings reveal how positive emotions during the pandemic are influenced by anti-pandemic technologies and measures, thus expanding current research (Martin et al., 2008; Song, 2017) in STS by revealing the complex impact of the large-scale implementation of biomedicine in society. Regenerative medicines produce hope for patients with ALS, and this hope works as a force to deal with uncertainties, making possible the suspension of diagnostic death sentences, destabilizing bureaucratic arbitrariness, and providing potential for the future (Song, 2017). Similarly, the hope generated by viral tests is a weapon against uncertainties and risks during the pandemic. It has been argued that the booming umbilical cord blood stem cell banking trend worldwide is associated with the capitalization of hope driven by future promissory economics (Martin et al., 2008). The RT-PCR industry is booming in China, and determining whether the hope related to viral tests contributes to the reproduction of capital requires further investigation. This study reveals that viral tests also assemble and mobilize trust in the government and its pandemic response. People’s trust could be facilitated by the implementation of mass testing campaigns. This research finding suggests that trust influences individuals’ risk perception toward infections and disasters (Ye and Lyu, 2020), risk responses (Zinn, 2008, 2016), and participation in mass testing (Xin et al., 2022), and it is also affected by practices of mass testing.

This research illustrates how public participation in health-protective behaviors and anti-pandemic measures is emotionally driven and influenced by technical, cultural, and political factors. It shows how the public accepted mass testing in mainland China during the first wave of the Delta variant outbreak and how different actors such as RT-PCR technology, lockdown policies, media representation, waiting times, and medical staff influence public attitudes toward mass testing. These findings are partially consistent with another recent study, which examines multi-dimensional factors influencing the public’s participation in a free population-wide testing program in Hong Kong (Xin et al., 2022). Specifically, this study also reveals that residents with a high level of perceived risk of infection trust the efficacy of mass testing programs in cutting off virus transmission and believe RT-PCR brings them certainty and comfort. They actively participate in mass screenings. The present study also explores how media representations, public health policy, and the process of mass testing can also influence individuals’ attitudes toward anti-pandemic measures; it thus suggests that cultural and political factors, as well as the larger social-political context, should be considered in any study of public responses to anti-pandemic measures.

The findings of this study lead to several practical suggestions for public health professionals when rolling out population-wide testing programs. First, measuring residents’ attitudes and perceptions of RT-PCR and mass testing is vital when planning population-wide screening programs. This study finds that the public’s perceived efficacy of RT-PCR and mass testing is associated with their trust in the technology of RT-PCR, emotional attachment to RT-PCR, perceived risk of infection, and consequences of infection on social and moral levels. The test’s perceived efficacy could influence their willingness to participate in mass testing (Xin et al., 2022). Since these multi-dimensional factors influencing public attitudes toward mass testing are associated with the effectiveness of mass testing—and since they might be different at different stages of the pandemic—they should be considered in policymaking, and pre-program research combining quantitative and qualitative methods is warranted. Second, effective implementation of mass testing and special arrangements for vulnerable groups of people may improve residents’ acceptance of massive viral tests. The findings demonstrate that easy access to testing points and effective sampling might improve people’s acceptance of compulsory testing, and vulnerable groups need to receive fast-track service when participating in mass testing. Good preparation for and management of screenings are vital, and information about the rationale, location, time slot, queue time, and process of mass viral tests should be clearly presented to the public (Xin et al., 2022). Convenient access to viral tests for vulnerable groups of citizens should be guaranteed to improve the overall coverage of testing.

Qualitative research methods can provide rich data and in-depth analysis for many research topics (Anderson, 2010). This qualitative study, which adopts an STS perspective, uses an inductive thematic analysis to examine the public’s attitudes toward mass testing during the COVID-19 pandemic and multi-dimensional factors shaping people’s participation in mass viral tests. However, a survey instrument that employs some classic health behavior models, such as the self-regulation theory of the common-sense model (CSM; Leventhal et al., 2016), the health belief model (HBM) (Janz and Becker, 1984), or the socio-ecological model (Bronfenbrenner, 1979), could provide validation to the research findings.

This qualitative research study has several strengths. First, the samples are heterogonous and include residents of different age ranges living in areas differently affected by the first wave of the Delta variant outbreak. The samples also include people living in areas under lockdown (Commodari and La Rosa, 2020; Chen et al., 2021) who had received viral tests multiple times and thus could have different perceptions of pandemic risk and responses to anti-pandemic measures. On the other hand, the potential limitations of the sample are that residents who tested positive are not included, and that data were collected during a single wave rather than multiple waves of outbreaks. The findings reported in this research are unique to this sample and the Guangzhou Delta variant outbreak; thus, avoiding overgeneralization of the research findings is necessary. Moreover, it would be relevant to further investigate a larger number of residents experiencing different waves of outbreaks in different areas. Adopting the theoretical perspective of ANT is another strength of the present study. Having been used previously in STS research (e.g., Martin et al., 2008; Stoopendaal and Bal, 2013), the utilization of the conceptual emphases of ANT in research is thought to reveal human and non-human actors along with multiple technological, cultural, psychological, and social factors and a more detailed description of their roles in shaping people’s attitudes (Latour, 2005). However, the interpretation of interview data, media reports, and online discussion could have been affected by the researcher’s experience and perception of anti-pandemic measures, although effort has been made to avoid misinterpretation by inviting several participants to discuss and verify the research findings during data analysis, article writing, and revision.

## Conclusion

This study focuses on pandemic responses during the first wave of the Delta variant outbreak in southern China, exploring how mass testing is perceived among ordinary people and identifying some critical factors that influence people’s attitudes toward mass testing policies. The study finds that residents actively participate in mass testing campaigns as mass viral tests are associated with hope and trust. The Reverse Transcription–Polymerase Chain Reaction experiment, negative test results, lockdown policies, queue times, medical staff, and media representations are all actors that shape people’s attitudes toward mass testing. The findings suggest that measuring residents’ attitudes and perceptions of RT-PCR and mass testing should be considered in policymaking. Effective implementation of mass testing and special arrangements for vulnerable groups of people may further improve residents’ acceptance of massive viral tests.

## Data availability statement

The raw data supporting the conclusions of this article will be made available by the authors, without undue reservation.

## Ethics statement

The studies involving human participants were reviewed and approved by the Survey and Behavioural Research Ethics Committee, The Chinese University of Hong Kong. The patients/participants provided their written informed consent to participate in this study.

## Author contributions

The author confirms being the sole contributor to this work and has approved it for publication.

## Conflict of interest

The author declares that the research was conducted in the absence of any commercial or financial relationships that could be construed as a potential conflict of interest.

## Publisher’s note

All claims expressed in this article are solely those of the authors and do not necessarily represent those of their affiliated organizations, or those of the publisher, the editors and the reviewers. Any product that may be evaluated in this article, or claim that may be made by its manufacturer, is not guaranteed or endorsed by the publisher.
